# Economic Costs of Delayed Diagnosis of Functional Motor Disorders: Preliminary Results From a Cohort of Patients of a Specialized Clinic

**DOI:** 10.3389/fneur.2021.786126

**Published:** 2021-12-08

**Authors:** Michele Tinazzi, Marialuisa Gandolfi, Stefano Landi, Chiara Leardini

**Affiliations:** ^1^Department of Neuroscience, Biomedicine and Movement Sciences, University of Verona, Verona, Italy; ^2^Department of Business Administration, University of Verona, Verona, Italy

**Keywords:** cost, diagnosis, health care delivery, rehabilitation, early intervention

## Abstract

**Background:** Functional motor disorders (FMDs) are prevalent and highly disabling conditions in young adults that can result in reduced independence. Despite advances in diagnosis and treatment, the economic burden of FMDs is largely unknown.

**Objective:** This pilot retrospective study provides a real-world overview of the economic costs related to delayed diagnosis of FMDs from a cohort of patients of a specialized clinic in Italy, based on Italian healthcare costs.

**Methods:** Sociodemographic data, clinical history, healthcare service utilization, and associated direct costs were collected for a period of up to 5 years before a definite diagnosis of FMDs in 40 patients.

**Results:** The mean time lag between the onset of FMDs symptoms and diagnosis was 6.63 years (±8.57). The mean annual use of recourses per patient was three specialist visits (95% CI 2.4–3.4) and three diagnostic examinations (95% CI 2.2–3.6) that made up a total of six investigations and over seven (95% CI 5.5–9.7) rehabilitation contacts per year per patient were used before a diagnosis of FMDs was established. In more than 50% of the cases, patients had been hospitalized or made an ER visit at least once before receiving the correct diagnosis. The average annual costs for delayed diagnosis, taking into account only direct healthcare costs (without medications), was about €2,302 (CI 95% €1,570–2,830) per patient [€1,524 covered by the NHS (CI 95% €1,214–1,834) and € 778 by the patient (CI 95% €606–960)]. Hospitalization accounted for €916 (CI 95% €670–1,160) per patient per year, followed by rehabilitation €493 (CI 95% €345–641) and diagnostic tests € 387 (CI 95% €314–460).

**Conclusion:** These preliminary results shed some light on the high healthcare services volume and direct healthcare costs from clinic to clinic for visits, unnecessary tests, and prescribed treatments in a real-world overview from a cohort of patients of a specialized clinic in Italy. It may represent a starting point for future studies to statistically test and quantify cost reduction after implementing appropriate healthcare pathways.

## Introduction

Functional motor disorders (FMDs) are part of a wide spectrum of functional neurological disorders (FNDs) [e.g., psychogenic non-epileptic seizures (PNES) and sensory and visual disturbances] characterized by abnormal movements (e.g., functional limb weakness, tremor, and dystonia) which can be altered by distractive maneuvers and are incongruent with movement disorders seen in typical neurological diseases (1, 2). Highly disabling non-motor symptoms (e.g., pain, fatigue, and anxiety) besides motor symptoms are also present (3). FMDs account for more than 50% of FNDs (prevalence 50/100,000 cases) mainly in young and middle-aged adults (35–50 years), in which they are a cause of disability and diminished quality of life (4) and a reason for leaving work or for reduced independence (5).

Over the past decade, despite advances in our understanding of the pathophysiology of FMDs (1, 6–8), clinical correlates (3, 9), and evidence-based treatment (8, 10), the illness is neither adequately diagnosed nor treated, as evidenced by the dissatisfaction expressed by neurologists (11) and by patients who feel misunderstood and abandoned by their healthcare providers (12). Such dissatisfaction reflects inadequacies within current clinical services for patients with FMDs (8, 12) and the lack of early diagnosis and disease-specific, evidence-based management. The difficulty in diagnosing and managing FMDs originates from a poor understanding of how symptoms are produced. The features that are generally associated with voluntary movement (distractibility and placebo resolution) have led neurologists to mistakenly think that simulation is the main mechanism underlying the disorders (1). Patients with FMDs differ from simulators, however, and report these disorders as involuntary and uncontrolled (6). The dissociation between the voluntary and the involuntary nature of FMDs perceived by patients stems from an altered perception of control of actions and their consequences (i.e., sense of agency) (7). Another crucial element is that FMDs have been conventionally considered secondary to emotional–psychological trauma (i.e., conversion disorders) (13). Recent epidemiological studies have demonstrated an overlap of stressful life events and psychological/psychiatric comorbidity between organic and functional disorders (14, 15). Accordingly, the causal role of psychological factors in FMDS has been removed from the Diagnostic and Statistical Manual of Mental Disorders (DSM V) where they are now defined as risk factors (2).

The literature emphasizes the importance of multidisciplinary three-stage stepped care to manage the phenomenological complexity of FMDs (16, 17). According to the Scottish model, the first step in successful management (step 1) is to diagnose FNDs symptoms early and appropriately explain them by a neurologist expert in the field. Step 2 entails brief therapy usually delivered by a physiotherapist for FMDs, and step 3 includes more complex multidisciplinary care involving the full rehabilitation team and psychiatry/psychology treatment. Wider acceptance of this stepped care model could enhance the care of patients with FMDs and reduce healthcare costs (17). The model is designed to guide healthcare providers, and its recommendations are not intended as prescriptive. In many cases, a revised approach will require (i.e., different national contexts and clinical frameworks) an established care pathway that is already working well.

Data from the first year (2018–2019) of the Italian Registry of Functional Motor Disorders (RI-FMDS) showed that the model is not currently operational in Italy: three physicians (range 1–25) on an average evaluated 80% of patients before a correct diagnosis was established, and about 73% of patients received one or more misdiagnoses of organic neurological disease (3, 9), with a diagnostic delay of about 6 years. Patients are often improperly referred to psychological and psychotherapy (rarely to physiotherapy) and leave their job due to an undiagnosed condition (3). The reasons for such poor management are grounded on two main issues: (1) an inappropriate referral to first-line specialists and lack of rehabilitation care (18).

There is limited information about health care costs in patients with FNDs because they are difficult to calculate, particularly for a complex disorder with a substantial delay to diagnosis (19, 20). Very recently, Stephen et al. (19) have provided national estimates of resource use and spending for FNDs in adults and children using national data on Emergency department and inpatient visits in the US in the period 2008–2017. They showed that patients with FNDs incurred total charges to the US health care system of more than $1.2 billion annually ($1.2 billion in adult FNDs and $88 million in pediatric FNDs), similar to those of neurological conditions that require high health care resource use.

Single-center studies on FNDs including a small sample of patients (from 25 to 73) have mainly focused on PNES and reported higher healthcare utilization due to a delay in correct diagnosis and appropriate patient management (20–24). No information on health care use and costs in FMDs patients is available although they represent the highest proportion of patients with FNDs.

In this work, we wanted to provide a real-world overview of the economic burden of FMDs from a cohort of patients of a specialized clinic in Italy before they had obtained a correct diagnosis. To do this, we defined medical resource consumption as the costs for specialist visits, diagnostic tests, emergency room (ER) visits, hospital admissions, and rehabilitation services in a period of up to 5 years before diagnosis, based on Italian healthcare costs (21). Estimation of utilization volumes and costs associated with delayed FMDs diagnosis can underline the importance of earlier diagnosis of FMDs for achieving better health outcomes, less unnecessary resource consumption, and lower costs. Adopting an early disease-specific care network model could improve the quality and the effectiveness of healthcare processes and bring savings to the national healthcare system.

## Methods

This pilot retrospective study involved 40 patients with a clinically definite diagnosis of FMDs (25), referred to the Parkinson's Disease and Movement Disorders Unit of Verona (Italy) and that were included in the Verona dataset of IR-FMDs (Department of Neurosciences, Biomedicine and Movement Sciences, University of Verona; Italian Academy for the Study of Parkinson's Disease and other Movement Disorders), satisfying the inclusion and exclusion criteria. Inclusion criteria were a detailed medical and medication history as documented by medical records or statements from informed relatives during a period of up to 5 years (3, 21); age ≥16 years; a clinically definite diagnosis of FMDs based on Gupta and Lang's diagnostic criteria (25); the presence of distractibility maneuvers and demonstration of positive signs (2); the presence of one (isolated FMDs) or more clinical motor symptoms (combined FMDs), including weakness, tremor, jerks, dystonia, gait disorders, parkinsonism, and facial motor disorders (3). Exclusion criteria were the presence of cognitive or physical impairments that precluded signing the informed consent form for participation in the study (3). For details see Table 1.

**Table 1:** Selection criteria, healthcare services, and direct cost description.

	**Description**
Inclusion criteria	Medical and medication history documented by medical records or statements from informed relatives during a period of up to 5 years (3, 21)
	A clinically definite diagnosis of FMDs based on Gupta and Lang's diagnostic criteria (25)
	Age ≥16 years
	The presence of distractibility maneuvers and demonstration of positive signs (2)
	The presence of one (isolated FMDs) or more clinical motor symptoms (combined FMDs), including weakness, tremor, jerks, dystonia, gait disorders, parkinsonism, and facial motor disorders (3)
Exclusion criteria	Cognitive or physical impairments precluding signing the informed consent form for participation in the study (3)
**Health care services and costs**	
Diagnostic test	Magnetic resonance imaging brain scan
	Neurophysiological tests
	Electroencephalogram
	Computed tomography brain scan
	Electrocardiogram
	Lumbar puncture
Specialist visits	General Neurologist
	Neurosurgeon
	Psychiatrist and psychologist
	Orthopedist
	Pain specialist
	Physical Medicine and Rehabilitation
	Rheumatologist
	Other
Emergency room visit (ERs)	
*Hospitalization*
Rehabilitation	Motor/neuromotor
	Psychotherapy
	Pain management
	Physical therapy
	Other

Sociodemographic information (age, sex, education level, and work status), clinical history (previous organic and non-organic diagnosis predating the final diagnosis, number of examining physicians, diagnostic tests), and clinical manifestations (isolated/combined FMDs, associated FNDs, psychiatric comorbidities, neurological comorbidities, and childhood predisposing factors) were collected.

### Healthcare Services Volume

The patients were invited to complete a questionnaire in March 2021. In the absence of a validated instrument for FMDs, we developed a customized questionnaire for collecting resource use data (26). A set of health services contributing to FMDs direct medical costs were identified by our previous study (3) and included in the questionnaire in form of questions. Patients were required to indicate only health services resources used because of the neurological symptoms. Patients were asked to self-report the number of units utilized for each type of service and provide the supporting documents released by the Hospital/ Institution, where possible diagnostic and utilization data were also matched with data from the hospital electronic medical record (EMR). Moreover, the year of the health service utilization preceding the correct FMDs diagnosis wase recorded. Moving from the health service volumes, the relative costs were calculated using official data from the Italian Ministry of Health for inpatients services (diagnosis-related group weighs) and Veneto Region (*Nomenclatore Tariffario Prestazioni Specialistiche Ambulatoriali*—Tariff Nomenclator for Specialist Outpatient Services) for outpatient services.

The total number of healthcare services consulted in the period of up to 5 years before the diagnosis of FMDs was retrospectively collected. If the disease duration was more than 5 years, we set a limit of 5 years because the estimations would not be accurate for supporting documentation that might be missing. In cases of a duration ≤ 5 years, all years were considered. Patients were asked to self-report the number of units utilized for each type of healthcare service because of the neurological symptoms and to provide supporting documentation. Wherever possible, diagnostic and utilization data were gleaned from the patient's medical and medication history as documented by medical records or statements from informed relatives in the period of up to 5 years. The estimation of the number of services utilized is conservative because only data proved by documents or hospital EMR were used. The year of healthcare service utilization preceding the establishment of correct FMDs diagnosis was recorded with the aim to find out if the use of services increases year after year or was steady.

Healthcare services were categorized into five groups: (1) diagnostic tests (i.e., magnetic resonance imaging (MRI) brain scan, neurophysiological tests, electroencephalogram, computed tomography scan, and lumbar puncture); (2) specialist visit (general neurologist, neurosurgeon, psychiatrist, psychologist, orthopedist, pain specialist, physical medicine and rehabilitation specialist, and rheumatologist); (3) emergency room (ER) visits; (4) hospital admission; (5) rehabilitation (motor/neuromotor, psychotherapy, pain management, physical therapy). Categories (1) and (2) may be conflated under the testing category, i.e., healthcare services that try to arrive at the root of the health problem. For details see Table 1. The definite diagnosis established by a neurologist expert in FMDs (MT) was reported in the questionnaire. The resources consumed for utilization of the healthcare service that established the definite diagnosis were excluded from volume and cost analysis.

### Direct Healthcare Costs

Two main categories of cost are relevant for the estimation of the economic burden of missed diagnosis of disease. Direct costs refer to the amount of money spent directly on treating or managing a disease. Direct costs comprise healthcare and non-healthcare costs. The former is defined as medical care expenditures related directly to the consumption of examinations, medications, medical visits, hospital admissions, ER visits, etc., whereas the latter refers to the use of non-healthcare resources such as transportation, household expenditures, and informal care of any kind (27). Indirect costs refer to productivity losses due to morbidity and mortality of the patient or the family involved as caregivers that are incurred in addition to the tangible (financial) costs of an illness [for example, the amount of time taken off (paid) work to seek medical attention and the loss of future expected income] (28).

For this work, we estimated the direct medical costs for utilization of each of the five healthcare service categories based on Italian healthcare reference rates. Cost of medications, non-healthcare direct costs, and indirect costs were excluded from analysis because the estimation might not have been accurate due to missing supporting documentation.

Direct cost estimates were divided into two categories: (1) costs to the patient and (2) costs to the national health service (NHS). Costs to the patient were defined as the total cost of out-of-pocket expenses for private healthcare services. The unit cost for these services was estimated by contacting five private medical centers and clinics across Veneto Region. Direct healthcare costs of hospital admission and outpatient services provided by the NHS during the study period were calculated using diagnosis-related group (DRG) weights and specific outpatient tariffs. These cost estimates were based on data from the Italian Ministry of Health (https://www.salute.gov.it/imgs/C_17_pubblicazioni_1094_allegato.pdf; https://bur.regione.veneto.it/BurvServices/Pubblica/Download.aspx?name=61_allegato_178934.pdf&type=9&storico=False).

Then the number of health service contacts per patient per year reported over 5 years was averaged to estimate the mean number of contacts that an FMDs patient is expected to have over 1 year. To estimate healthcare care costs, the average number of contacts was then multiplied by the unit cost for each contact reported and by the number of FMDs patients observed in our study in a given year. Finally, the mean annual cost per patient before the definite diagnosis was obtained to estimate the economic impact of the missed diagnosis.

### Statistical Analysis

Statistical analysis was performed using SPSS Statistics 19.0 (IBM SPSS Statistics for Windows, Version 19.0. Armonk, NY: IBM Corp.) The confidence intervals were estimated by bootstrapping the data 1,000 times. Since data distribution violates normality assumptions (Shapiro-Wilk test: *p* < 0.05) non-parametric analyses were performed. Kruskal–Wallis tests were employed to determine differences in costs between groups.

### Patient Informed Consent and Ethical Approval

The study was incorporated within the Verona dataset of RI-FMDs, and so informed consent for participation in the registry sufficed. Approval was obtained from the local Institutional Ethics Committee (Project Number 132 1757CESC).

## Results

### Sociodemographic and Clinical Data

Table 2 presents the sociodemographic, clinical history, and clinical manifestations of FMDs for this sample, the majority of which were women (83%) and employed (54%). The mean time lag between the onset of symptoms and definite diagnosis of FMD was 6.63 years (±8.57); the time lag was >5 years in 10 patients (25%). Most (65%) were affected by the combined phenotype (i.e., weakness + tremor) or other FNDs (26%). Psychiatric comorbidity (mainly depression and anxiety) was documented in 30%, whereas neurological comorbidities were noted in 33%, consistent with literature (3, 29).

**Table 2:** Sociodemographic data, clinical history, and clinical manifestations of FMDs.

**Sociodemographics**		
Patients, no.	40	
Sex, female, no. (%)	33	(83)
Age, yrs, mean (SD)	41	(15.12)
Education, yrs, mean (SD)	10.75	(3.76)
Work status, no. (%)	39	(97.5%)
Employed	21	(54)
Unemployed	5	(13)
Other (housewife, student, retired)	13	(33)
**Clinical history**		
**Previous incorrect diagnosis, yes/no. (%)**	**21**	**(53)**
Dystonia	1	(5)
Parkinson disease/Parkinsonism	2	(10)
Cerebrovascular disease	1	(5%)
Tremor	2	(10)
Seizure	2	(10)
Neuroinflammation	2	(10)
Other	13	(62)
Previous medical visits before correct diagnosis, no. (%)	31	(78)
Number of medical visits before correct diagnosis, mean (SD)	5.19	(6.04)
**Clinical manifestations of FMDs**		
**The time lag between symptom onset and FMDs diagnosis**, years, mean (SD)	**6.63**	**(8.57)**
**Isolated FMDs, no. (%)**	14	(35)
Weakness	7	
Tremor	1	
Dystonia	1	
Myoclonus	1	
Gait disorders	3	
Other	1	
**Combined FMDs, no. (%)**	26	(65)
**Associated FND, no. (%)**	26	(65)
Non-epileptic seizure	8	
Visual	6	
Cognitive	8	
Sensory	21	
Pain (fibromyalgia)	6	
Irritable bowel syndrome	2	
**Psychiatric comorbidities, no. (%)**	12	(30)
Major depression	8	
Anxiety	6	
Dissociative fugue	1	
Somatization	2	
Eating disorders	1	
**Neurological comorbidities, no. (%)**	13	(33)
Multiple sclerosis	1	
Parkinson's disease	1	
Neuropathy	3	
Seizure	3	
Migraine	4	
**Other**	**4**	
**Childhood predisposing factors, no. (%)**	**8**	**(20)**
**Psychological trauma**	**5**	
**Physical trauma**	**4**	
**Both**	**1**	

*No., number; yrs, years; %, percentage; SD, standard deviation*.

### Healthcare Service Utilization Volume

Table 3 presents the volume of healthcare services utilization.

**Table 3:** Resource utilization and costs in euros.

**Estimation of resources utilization and costs**	**Volumes**		**Total cost (€)**		**Patients Cost (€)**		**NHS Cost (€)**	
	**No**.	**Mean (±SD)**	**Total Cost**	**Mean (±SD)**	**Total Cost**	**Mean (±SD)**	**Total Cost(%)**	**Mean (±SD)**
**Diagnostic tests**	315	7.88 (6.07)	41,500	1,037 (840)	12,013	301 (475)	29,499	737 (705)
**Specialist visits**								
Total	310	7.75 (6.8)	29,059	726 (840)	25,820	645 (588)	3,239	81 (111)
General Neurologist	152	3.8 (3.5)	14,237	356 (348)				
Neurosurgeon	14	0.35 (0.86)	1,206	31 (95)				
Psychiatrist and Psychologist	40	1 (1.72)	3,495	88 (133)				
Orthopedist	24	0.6 (1.4)	2,248	56 (133)				
Pain specialist	19	0.48 (1.29)	1,952	49 (125)				
Physical Medicine and Rehabilitation	15	0.38 (1.10)	1,301	33 (92)				
Rheumatologist	18	0.45 (1.63)	1,732	44 (168)				
Other	28	0.7 (1.55)	2,888	72 (153)				
**ERs**	73	1.83 (3.02)	19,140	478 (766)			19,140	478 (766)
**Hospitalization**	30	0.75 (0.78)	98,041	2,451 (2,468)			98,041	2,451 (2,468)
**Rehabilitation**								
Total	817	20.45 (31.9)	52,792	1,319 (2.360)	44,182 (54)	1,104 (2,185)	8,610	215 (839)
Motor/neuromotor	302	7.55 (24.7)	19,182	479 (1,670)				
Psychotherapy	260	6.50 (13.9)	18,551	463 (1,109)				
Pain management	22	0.55 (1.65)	1,810	46 (123)				
Physical therapy	230	5.75 (11.2)	12,550	313 (551)				
Other	3	–	–	–				
**Total**			240,544 (100)	5,987 (4,270)	82,015	2,050 (2,862)	158,529	3,963 (2,847)

*No., number; ERs, emergency room services % share of cost category on total cost; NHS, National Health Service; SD, standard deviation; €, euros*.

The total amount of health services contacts for the 40 patients were of 1,545. The most amount was registered for investigation (specialist visits and diagnostic examinations) and rehabilitation services. On an average, 7.7 specialist visits (95% CI 5.64–9.86) and 7.8 diagnostic examinations (95% CI 6–9.7) made up a total of 15.5 tests before a diagnosis of FMDs was established (range, 11–20). The most frequent specialist visit was consultation with a general neurologist [mean 3.8 (95% CI 2.7–4.8)], followed by a psychiatrist or a psychologist. In all, non-neurologist visits accounted for 47% of specialist visits [mean 3.95 (95% CI 2.7–5.2)]. There was a marked variability in the use of rehabilitation services: on an average, 20 rehabilitation visits (95% CI 10.6–30.3). Motor and neuromotor rehabilitation was most frequent (37%), followed by psychotherapy (32%) and physical therapy (28%). ER visits ranged from a mean of 1.8 in one patient (95% CI 0.9–2.8) to two visits in 25% of the patients with multiple ER visits, and one hospital stay (95% CI 0.5–1) before diagnosis of FMDs was established: in more than 50% of the cases, the patient had been hospitalized or made an ER visit at least once before receiving the correct diagnosis.

Table 4 shows the mean annual use of resources per patient. On an average around three specialist visits (95% CI 2.4–3.4) and three diagnostic examinations (95% CI 2.2–3.6) made up a total of six investigations per year per patient before a diagnosis of FMDs was established. Moreover, over seven (95% CI 5.5–9.7) rehabilitation contacts per year per patient were used before diagnosis.

**Table 4:** Mean annual cost and volumes per patient.

**Estimation of resources utilization and costs**	**Annual contacts per patient**		**Annual cost per patient (€)**	
	**Mean (±SD)**	**95% CI**	**Mean (±SD)**	**95% CI**
Diagnostic tests	2.9 (2.7)	(2.4–3.4)	387 (386)	(314–460)
Specialist visits	2.9 (3.5)	(2.2–3.6)	271 (325)	(209–333)
ERs	0.7 (1.5)	(0.4–1.0)	178 (370)	(108 −248)
Hospitalization	0.3 (0.5)	(0.2–0.4)	916 (1,300)	(670–1,160)
Rehabilitation	7.6 (11.1)	(5.5–9.7)	493 (779)	(345 −641)
Total			2,302 (2, 010)	(1,570–2,830)

*CI, confidence interval; SD, standard deviation; specialist visits include general neurologist, neurosurgeon, psychiatrist and psychologist, orthopedist, pain specialist, physical medicine and rehabilitation, rheumatologist, other; ERs, emergency room services; €, euros; SD, standard deviation*.

### Direct Healthcare Costs

**Figure 1:**
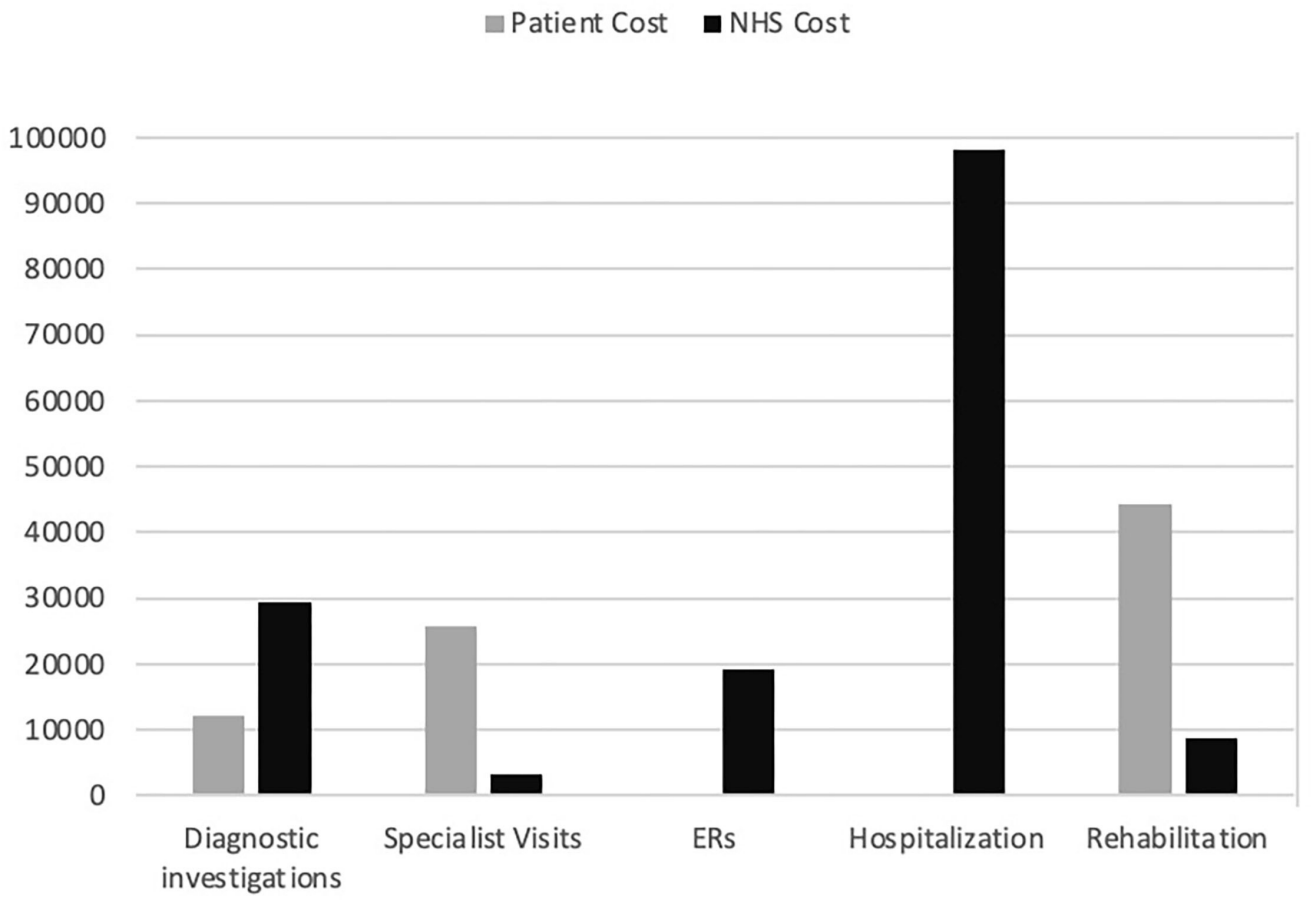
Total cost in euros to the patient and to the NHS by healthcare services group. Diagnostic investigations: magnetic resonance imaging brain scan, neurophysiological tests, electroencephalogram, computed tomography scan, electrocardiogram, lumbar puncture; Specialist visit: general neurologist, neurosurgeon, psychiatrist, psychologist, orthopedist, pain specialist, physical medicine and rehabilitation specialist, rheumatologist; ERs: emergency room visit; Hospitalization: hospital admission; Rehabilitation: motor/neuromotor, psychotherapy, pain management, physical therapy; NHS, national health service.

**Figure 2:**
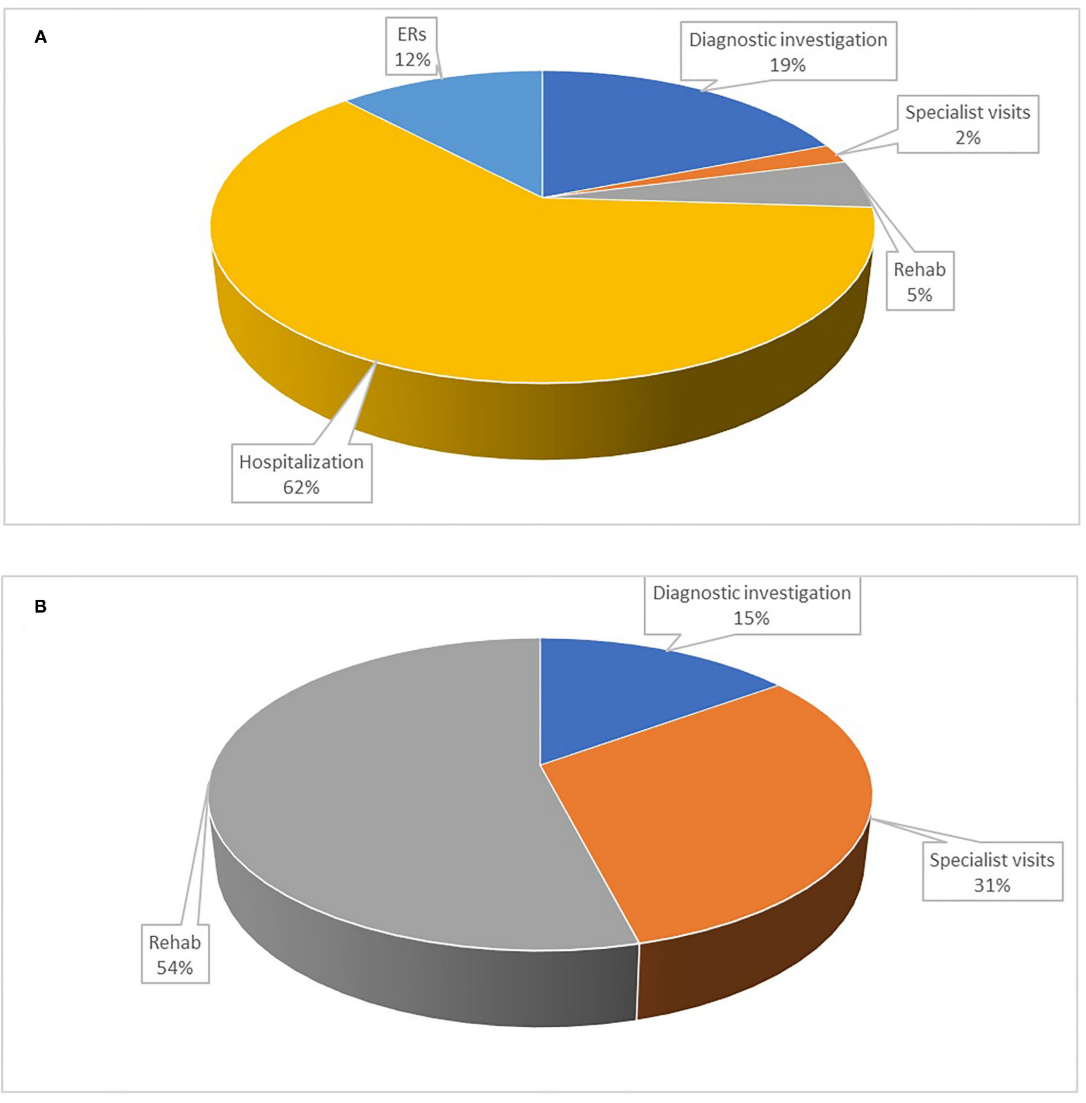
Share of costs in percentage by healthcare service for the NHS and the patient. **(A)** Costs for the NHS; **(B)** Costs for the patient. Diagnostic investigations: magnetic resonance imaging brain scan, neurophysiological tests, electroencephalogram, computed tomography scan, electrocardiogram, lumbar puncture; Specialist visit: general neurologist, neurosurgeon, psychiatrist, psychologist, orthopedist, pain specialist, physical medicine and rehabilitation specialist, rheumatologist; ERs: emergency room visit; Hospitalization: hospital admission; Rehab: motor/neuromotor, psychotherapy, pain management, physical therapy.

The total direct healthcare cost for the sample was €240,544 (Table 3). Figure 1 shows the total cost distribution based on the five categories of healthcare service and by patient/NHS costs. The main drivers of cost were hospitalization (40%), rehabilitation (22%), diagnostic tests (17%), ER visits (10.5%), and specialist visits (10.5%). The distribution of costs by category differed between NHS and patients (Figure 2), with the latter mainly taken up by rehabilitation (54%) and specialist visits (31%). The major cost categories for the NHS were ER visit/hospital admission (74%) and diagnostic testing (19%).

The average annual costs for delayed diagnosis, taking into account only direct healthcare costs (without medications), was about €2,302 (CI 95% €1,570–2,830) per patient per year of delay in diagnosis [€1,524 covered by the NHS (CI 95% €1,214–1,834) and € 778 by the patient (CI 95% €606–960)]. Hospitalization accounted for €916 (CI 95% €670–1,160) per patient per year, followed by rehabilitation €493 (CI 95% €345–641) and diagnostic tests € 387 (CI 95% €314–460).

Regarding the distribution of costs over the 5 years before the establishment of a definite diagnosis of FMDs, resource consumption tended to stabilize over time (Table 5). The per patient per year cost is constant over years. The only healthcare service category with a statistically significant change was the five-fold increase in ER visits in the last 2 years before FMDs diagnosis (Table 6). Given that the mean time to diagnosis was 6 years, we can estimate an average cost of delayed diagnosis of around €13,812 per patient (€9,144 incurred to the NHS; €4,669 to the patient). Using FMDs prevalence (5), the estimated amount of annual direct healthcare cost for not diagnosed patients with FMDs of the Italian population is €34.5 million (22.5 covered by NHS and 11.5 by patients).

**Table 5:** Mean annual cost per patient during the time to diagnosis in euros.

**Years to diagnosis**	**Annual cost per patient (€)** ^**§**^	
	**Mean (±SD)**	**95% CI**
5	2,286 (2,524)	(636–3,940)
4	1,684 (1,481)	(846–2,520)
3	1,673 (1,527)	(925–2,420)
2	2,485 (2,148)	(1,730–3,240)
1	2,345 (2,104)	(1,690–3,010)
Mean annual cost per patient	2,302 (2,010)	(1,570–2,830)

*SD, standard deviation; CI, confidence interval; €, euros*;

§ *Kruskal–Wallis test: 1.244 (p = 0.81)*.

**Table 6:** Annual cost in euros per patient.

	**Annual total cost per patient (€)^**§**^** **Mean (±SD)**	**Annual ER cost per patient (€) ^**§§**^** **Mean (±SD)**
In the last 2 years before diagnosis	2,407 (2,109)	210 (439)*
Previous years before diagnosis	1,821 (1,755)	40 (126)*

*SD, Standard deviation; €, euros*;

§ *Kruskal–Wallis test 2.41 (p = 0.143)*;

§§ *Kruskal–Wallis test 4.25 (p = 0.04)*;

* *p < 0.05*.

## Discussion

With this study, we explored the volume and the direct medical costs of delayed diagnosis of FMDs in a cohort of patients during a period of up to 5 years before they received correct diagnosis and management at a specialized tertiary clinic in Italy. The direct medical cost before diagnosis was estimated at €2,302 per patient per year, divided into €1,524 to the NHS and €778 to the patient for out-of-pocket expenses. Major cost drivers were hospitalization (40% of total costs), rehabilitation services, and diagnostic testing. The costs of diagnostic tests and hospitalization are direct costs for the NHS, whereas the costs for specialist visits and rehabilitation services are covered by the patient.

A recent study assessing US emergency department (ED) and inpatient use and charges for FNDs using national data in the period 2008–2017 underlies the importance of increasing information on health care and costs in patients with FNDs, pointing out that unnecessary investigations and iatrogenic harm inflate costs in the face of neglected rehabilitative treatments (19). Previous literature focused on single-center studies on PNES (20–24) showing a considerable burden of healthcare utilization costs due to a delay in the correct diagnosis, unnecessary therapy, and iatrogenic consequences. Our study cannot be compared with this previous literature due to differences in the patients' selection (adults and pediatric with FNDs and adults with PNES), methodology used in retrospectively retrieving economic data (International Classification of Disease codes, questionnaires, EMR), and settings (emergency department, inpatient use, single medical centers). However, in our pilot single-center retrospective study on a small cohort of patients with FMDs from a specialized clinic in Italy, we reported for the first time evidence of considerable direct healthcare volumes and costs before the correct diagnosis also in patients with FMDs.

In our work, the mean time lag between the onset of symptoms and FMDs diagnosis (6.63 years) is in line with previous studies (3, 20, 21). Often inappropriate procedures, such as psychotherapy for example, translated into a steady additional cost year by year until a definite diagnosis was established.

These preliminary results shed some light on the high healthcare services volume and direct healthcare costs of FMDs patients from clinic to clinic for visits, unnecessary tests, and prescribed treatments (3, 19). Most (78%) reported multiple consultations and numerous diagnostic tests before they received a correct diagnosis of FMDs. A plausible explanation is “doctor shopping,” which we believe reflects issues associated with the diagnosis of FMDs, including miscommunication between physicians and patients, a reluctance/failure to accept the diagnosis, and the lack of a therapeutic plan and clear treatment goals (3, 18, 30, 31). Before diagnosis, many specialist visits involved neurologists first, then psychiatrists. Around half of all specialist visits were not a consultation with a neurologist, but rather a psychologist or a psychiatrist. This indicates a lack of recognition of the disorders by neurologists and other healthcare providers who wrongly referred the patient for inappropriate and unnecessary diagnostic testing and specialist visits (“doctor shopping”). The same problem concerns the type of therapy and treatment: only 37% of the patients underwent rehabilitative treatment (motor/neuromotor), which was ineffective because it did not address the physiopathology of the disorder. This phenomenon is further confirmed by the reported incorrect use of rehabilitation services, where psychotherapy accounted for 32% and physical therapy 28% of the services delivered. In brief, the diagnostic delay generates unjustified costs for diagnostic testing, ineffective treatment, and hospital admission. Referring to an FMDs specialist would reduce the latency to diagnosis and lower cost, thereby avoiding unnecessary multiple consultations and tests (3, 30, 31).

Moreover, on an average the cost of health services used is steady year after year with no peak for any services except ER. In the 2 years before the establishment of correct diagnosis, the data show repeated ER visits and hospital admission when the diagnosis is finally made (the use and costs of the last hospitalization have not been included in resource analysis). The data show that because primary healthcare services do not solve the problem, patients are admitted to the hospital where costs are higher. Considering only direct medical costs (excluding medications) and an average delay of 6 years, the total cost before diagnosis is around €14,000 per patient. The total cost is a conservative estimation because, to avoid recall bias, we reported only resource use data that could be confirmed by documents or hospital EMR. Actually, we calculated only direct medical costs, excluding costs for medications and non-healthcare direct and indirect costs. Moreover, costs do not take into account the costs of medications, transportation, and caregiving and, in particular, the cost of informal care and the loss of productivity due to time taken off (paid) work. While the cost of medications may not be relevant compared to PNES, for example, FMDs still incur considerable indirect costs.

Obtaining earlier diagnosis, increasing health professionals' knowledge of FMDs can improve health outcomes and reduce unnecessary resource consumption and costs. Due to its engendered costs, the economic burden of illness is acutely underexplored, yet essential for public health policy makers.

This work is the first which would attempt to fill this gap when healthcare systems are increasingly under pressure to enhance the choice of value for money in healthcare programs. The Scottish three-stage stepped care model constitutes a reference in offering healthcare providers guidance to deal with FNDs. However, it needs to be tailored to the specific cultural and NHS framework of the Italian context.

First, early and thorough assessment by healthcare providers and by general practitioners (GPs) (primary care) is needed to refer the patient toward correct diagnosis as soon as possible (18). Educational efforts should involve all professional categories (i.e., GPs, physiatrists, psychiatrists, physiotherapists, psychologists) and all levels of healthcare professional training and continuing education (i.e., undergraduate and post-graduate education pathways) to share knowledge on the pathophysiology, clinical correlates, and management principles of FMDs (8, 18). This would aid in the development of shared (partially overlapping) expertise and catalyze interdisciplinary management of FMDs.

Second, patients should be promptly referred to a neurologist expert in FMDs (or to a general neurologist who can refer the patient to an experienced neurologist) for correct diagnosis and communication, and to triage the patient toward the most suitable treatment according to available options and phenomenological complexity, including physiotherapy, skills-based psychotherapy, and/or multidisciplinary interventions (8, 10, 32, 33). Rehabilitation followed by home-based management can play a crucial role in the multidisciplinary management of these patients through education, movement retraining, and self-management strategies in a positive and a non-judgmental context (8, 10, 32, 33).

Finally, complicated patients with combined FMD phenotypes (3), paroxysmal symptoms, and psychiatric comorbidities or overlapping organic neurological disease (9) may require a further third-line interdisciplinary and multidisciplinary approach (16). More advanced diagnostic testing and specialist visits might be focused on evaluating only organic symptoms paroxysms, thus averting a waste of unnecessary healthcare resources.

Scattered evidence suggests that rehabilitation might further decrease healthcare costs by reducing symptom severity and improving quality of life (34, 35). Nielsen et al. (2017) found an improvement in motor symptoms in 72% of the patients attending physiotherapy (*n* = 30) compared with 18% of patients who did not (*n* = 30) over 6 months (34). The improvement was associated with moderate-to-large treatment effects across various clinical outcomes, such as quality of life as measured by Short Form 36. In addition, this feasibility study showed that rehabilitation had a high probability of being cost-effective, with a mean incremental cost per quality-adjusted life-year gained at £12,087 (34).

Whether disease-specific multidisciplinary three-stage stepped care is truly cost-effective needs to be explored by cost-effectiveness studies. The results of this work are an initial step to determine whether reducing unnecessary medical testing through accurate and early diagnosis followed by patient-centered multidisciplinary care can improve patient outcomes and save health costs.

The main strength of our study is that for the first time the economic burden of FMDs has been estimated in a cohort of patients with a clinically definite diagnosis made at the same clinic for movement disorders. The analysis of health services resources was used because neurological symptoms showed that the main drivers of utilization volume and cost are the broad categories of services, testing, rehabilitation, and hospitalization, as well as the specific type of service (neurology, psychology, etc.). The variety of services reflect the patient's difficulties in seeking a definite diagnosis that may take up to 5 years before it is established.

Our data should be interpreted cautiously given the small sample size, also including patients with FMD with comorbid organic neurological and psychiatric disorders, which may have lengthened time to diagnosis and increased healthcare costs. However, our cohort's proportion of these comorbidities is consistent with those reported in a larger sample of FMD patients (3, 9, 29), thus indicating that our patient's sample is representative. Data from a larger sample of FMDs stratifying patients with and without neurological/psychiatric comorbidity would generalize the present results.

Other significant limitations of this work are the lack of an instrument designed/validated for assessing costs in FMDs and the lack of a control group with “organic” motor disorders hampers having a typical healthcare expenditure to compare with. The lack of patient-reported outcome measures and reported experience measures does not allow us to assess the patients' dissatisfaction about their pathway care. Moreover, the retrospective design did not allow us to retrieve indirect medical costs or evaluate informal care and productivity loss for high recall bias risks. Finally, we could not determine the severity of the recorded symptoms and quality of life as we did not employ an instrument for rating them (i.e., simplified functional movement disorders rating scale and SF36).

Notwithstanding these limitations, our study provides a starting point for future studies to statistically test and quantify cost reduction after implementing appropriate healthcare pathways to analyze an economic comparison between a diagnostic protocol in practice vs. none. The goal is to understand the economic burden of undiagnosed FMDs and the magnitude of increasing/decreasing cost overtime before/after diagnosis.

Future studies would (1) validate the questionnaire specifically for patients with FMDs to improve the quality of resource use data generated, (2) evaluate whether there exists meaningful differences in cost between different phenotypes, and (3) consider the number of individual providers, as opposed to specialist visits, to detail how many different opinions motor FMDs might have.

## Data Availability Statement

The raw data supporting the conclusions of this article will be made available by the authors, without undue reservation.

## Ethics Statement

The studies involving human participants were reviewed and approved by Ethics Committee Verona and Rovigo Province (CESC) (Project Number 132 1757CESC). Written informed consent to participate in this study was provided by the participants' legal guardian/next of kin.

## Author Contributions

MT: design, conceptualization and execution of the study, review, and critique of the statistical analyses and manuscript. MG: execution of the study, acquisition of clinical data, drafting the manuscript, and review and critique of the manuscript. SL: execution of the statistical analyses, drafting the manuscript, and review and critique of the manuscript. CL: design, conceptualization of the study, review and critique of the statistical analyses and manuscript. All authors contributed to the article and approved the submitted version.

## Conflict of Interest

The authors declare that the research was conducted in the absence of any commercial or financial relationships that could be construed as a potential conflict of interest.

## Publisher's Note

All claims expressed in this article are solely those of the authors and do not necessarily represent those of their affiliated organizations, or those of the publisher, the editors and the reviewers. Any product that may be evaluated in this article, or claim that may be made by its manufacturer, is not guaranteed or endorsed by the publisher.
